# Reverse-mentorship of the core concepts in philosophy and mental health: a medical education case report

**DOI:** 10.1192/bjo.2021.402

**Published:** 2021-06-18

**Authors:** Michael Jewell, Manzar Kamal, Richard Bayney, Heidi Hales

**Affiliations:** West London NHS Trust

## Abstract

**Aims:**

The aim of this medical education case report was to outline the development and outcomes of a reverse-mentorship project that enabled cross-generational collaborative learning. The project took the shape of a philosophy of psychiatry journal club facilitated by a psychiatry core trainee in west London, UK.

**Background:**

Reverse-mentorship reverses traditional roles of mentor and mentee. It is an increasingly fashionable concept in medical education. The junior mentors the senior clinician. The implicit learning outcomes include provision of a two-way learning process, development of mentoring skills for the more junior clinician and collaboration that builds social capital within the workplace. Reverse-mentorship is effective when the junior mentor is recognised for their expertise in a particular area. In this instance, the junior mentor has a special interest in the philosophy of psychiatry.

**Method:**

Junior mentor and senior mentees formed a monthly journal club. The club tracked arguments from anti- and biological psychiatry on the meaning of mental illness. The debate offered insight into a semantic analysis of mental illness and a deeper conceptual understanding of medicine. The learning material derived from the core concepts of philosophy and mental health (Fulford et al.). The role of the mentor was to facilitate group discussion around arguments from relevant papers. A survey, adapted from a recent reverse-mentorship review article, measured the quality of educational experience for mentor and mentees.

**Result:**

Overall, mentees (senior clinicians) agreed that the mentor (junior clinician) displayed attributes and behaviours for effective mentoring across most domains, including enthusiasm, effective communication, respect for mentee expertise and active listening to the needs of the mentee. The mentor was particularly impressed with the mentees’ openness to learn new concepts and respect shown. General reflections on the experience of reverse-mentorship were positive overall. A thematic review highlighted particular aspects, including: a good way to learn a new skill and great opportunity to develop professional skills of mentoring.

**Conclusion:**

The importance of mentoring in medical education is well established. Reverse-mentorship is a new concept that looks to harness the unique qualities of millennials, including their aptitudes for empowerment, innovation and collaboration. This medical education case report shows that an enthusiastic junior clinician can successfully pilot an educational-mentoring scheme aimed at senior clinicians. To make more explicit the intuitive benefits of reverse-mentorship, longitudinal reviews are needed. However, this case report contributes important insights into this burgeoning field of medical education.

